# District-level analysis of temperature-related excess mortality in the Netherlands

**DOI:** 10.1097/EE9.0000000000000534

**Published:** 2026-10-15

**Authors:** Aisha Ndiaye, Hein Daanen, Jan van de Kassteele, Kees de Hoogh, Laurens Zwakhals, Liselotte van Asten, Werner Hagens, Jochem Klompmaker

**Affiliations:** a National Institute for Public Health and the Environment (RIVM), Bilthoven, The Netherlands; b Department of Human Movement Sciences, Faculty of Behavioural and Movement Sciences, Vrije Universiteit Amsterdam, Amsterdam, The Netherlands; c Institute for Risk Assessment Sciences (IRAS), Utrecht University, Utrecht, The Netherlands

**Keywords:** Temperature, Excess mortality, Distributed lag nonlinear models, Case time series, Small area

## Abstract

**Background:**

While most epidemiological studies have assessed temperature–mortality associations at broad geographical scales, recent research highlights that temperature-related mortality risks are spatially heterogeneous due to local differences in exposure, vulnerability, and adaptive capacity. The aim of this study was to assess the impact of ambient air temperature on all-cause mortality at the district level in the Netherlands.

**Methods:**

A three-stage design was applied to assess the association between daily average air temperature and mortality across 3,116 districts and two age groups (0–74, 75+) for the period 2010–2019. First, a case time series design with distributed lag nonlinear models was used to estimate temperature–mortality associations for 355 Dutch municipalities. Second, municipality-level estimates were pooled using a multivariate meta-regression with municipality-level demographic, socioeconomic, climatological, and topographical predictors. Finally, the meta-regression model was used to downscale the risk estimates to the district level and to estimate standardized excess heat- and cold-related mortality rates.

**Results:**

Heat-related excess mortality was elevated in urban areas, whereas cold-related excess mortality was more pronounced in northern and southern districts compared with areas in the center of the Netherlands. During the study period, heat was associated with an average of 346 excess deaths per year (empirical 95% confidence intervals: 44–558), and cold with 11,988 excess deaths per year (7,554–15,609). This corresponds to standardized excess mortality rates of 2.2 (0.3–3.5) and 73.3 (46.8–94.7) per 100,000 person-years for heat and cold, respectively. The 75+ group showed more pronounced mortality risks compared with the younger age group.

**Conclusion:**

Our results indicate geographical heterogeneity in temperature-related standardized mortality rates, with high heat-related mortality in urban areas and more pronounced regional clustering of cold-related mortality. These findings can help local governments to implement climate adaptation measures to prevent heat- and cold-related excess mortality according to location-specific vulnerabilities.

**What this study adds**

By characterizing small-area-level temperature-related mortality burden, our study may help to inform public health policy in the Netherlands, and may support future assessments of temperature-related health risks under climate change. In addition, while our analysis focuses on the Netherlands, its strengths and relevance lie in the use of high spatial resolution exposure and health data, as well as the application of novel modeling techniques that enable the estimation of small-area mortality risks.

## Introduction

Extensive evidence indicates that short-term exposure to suboptimal ambient temperatures is associated with increased mortality risk. Globally, suboptimal temperature is associated with 9.4% of all deaths annually, of which 0.9% can be attributed to heat and 8.5% to cold.^1^ Due to climate change, extreme weather events such as heatwaves are expected to increase in the next decades both in frequency and intensity.^1–3^ Current projections indicate that heat-related mortality will increase under warmer climates.^4^ At the same time, while exposure to cold spells may decrease in the next decades, the vulnerable population is expected to increase due to population aging.^5,6^ Thus, adaptation to and preparedness for extreme temperatures remain crucial to reduce heat- and cold-related health burdens worldwide.^7,8^

In the past decades, epidemiological research has mostly focused on estimating temperature–mortality risks over broad geographical areas, for which exposure data and health outcomes are aggregated to provide sufficient statistical power.^9–12^ However, a growing body of literature has shown that the health impacts of temperature are spatially heterogeneous, as temperature exposure, population vulnerability, and capacity for climate adaptation can differ due to area-specific biophysiological, sociocultural, infrastructural, and technological factors.^13,14^ Consequently, small-area analyses are better suited to identify local vulnerabilities to temperature and calculate spatially detailed health impacts.^15,16^

The need to identify small-area level risks combined with the growing availability of data collected at fine geographical resolution has led to the development of new methodologies for small-area level epidemiological analysis.^17–20^ These methodologies have been successfully applied at the neighborhood level in England and Wales,^17^ at the municipality level in Norway,^18^ and at the pseudo-canton level in France.^19^ Studies conducted in the Netherlands have predominantly relied on country-aggregated time series data to evaluate temperature–mortality associations.^21–24^ Consequently, spatial contrasts in temperature exposure and in population vulnerability were largely overlooked, while possibly contributing to heterogeneity in temperature–mortality associations across the country.

The aim of this study was to assess the impact of ambient air temperature on all-cause mortality at a small-area level in the Netherlands, using the three-stage design proposed by Gasparrini et al^17^ to identify small-scale risk patterns. First, we estimated the association between daily average air temperature and all-cause mortality for all Dutch municipalities. Next, we pooled municipality-specific risk estimates in a multivariate meta-regression, using municipality-level demographic, socioeconomic, climatological, and topographical predictors. Finally, we applied the meta-regression model to downscale the risk estimates to the district level, and we assessed excess heat- and cold-related standardized mortality rates. By employing this novel study design with two levels of analysis (from municipalities to districts), we were able to analyze data and estimate health impacts at the highest possible resolution, without having to build separate models for each district—which would not have been feasible given the low number of deaths in each area. By characterizing the temperature-related excess mortality at a small-area level, this study can support policy-makers in prioritizing temperature-related adaptation measures and tailoring public health interventions.

## Methods

### Study area and period

The Netherlands has a temperate maritime climate influenced by the North Sea and is relatively flat. In 2019, the population was around 17.3 million inhabitants, over an area of approximately 41,543 km^2^. The country has 355 municipalities, and on average, a municipality consists of nine districts. In 2019, the number of inhabited districts was 3,116. On average, districts had an area of 11.18 km^2^ (median = 6.49 km^2^) and a population of 5,544 residents. The study period 2010–2019 was selected to avoid potential confounding effects of the coronavirus disease 2019 (COVID-19) pandemic and because prior epidemiological research conducted in the Netherlands identified differences in temperature-related mortality risk before and after the first activation of the Dutch national heatwave plan in 2010.^24^

### Mortality and exposure data

We collected all-cause mortality cases between 1 January 2010 and 31 December 2019 from Statistics Netherlands.^25^ Based on the individual mortality cases, we created district-specific daily time series of all-cause mortality counts for 3,116 districts within 355 municipalities in the Netherlands. Daily temperature data were obtained from a high-resolution spatiotemporal model developed and validated by Bussalleu et al.^26^ Daily mean, minimum, and maximum ambient temperatures were estimated across Europe for the 2003–2020 period using a two-stage hybrid modeling approach. Briefly, daily ambient temperature 2 m above ground level was predicted at a fine spatial resolution of 1 × 1 km, combining satellite data, station-based temperature measurements, atmospheric re-analysis data, and land-use information.^26^ For the present analysis, we derived district-specific daily mean temperature values for the 2010–2019 period by overlaying the gridded temperature data with the 2019 administrative district boundaries. For each district, we calculated the area-weighted average of the daily mean temperatures of all grid cells intersecting its borders, with weights proportional to the grid cell–district intersection area.

In addition, for the purpose of a sensitivity analysis, we obtained daily temperature data from the ERA5 re-analysis dataset.^27^ The ERA5 dataset provides hourly and daily historical climate and weather data estimated on a 28 × 28 km scale using an integrated forecasting system.^28^ The parameter for surface temperature corresponds to the temperature of air at 2 m above the surface of land, sea, or inland waters. District-specific daily mean temperature values were derived using the same area-weighted averaging approach, based on the grid cells intersecting each district.

### Meta-predictors

We compiled a dataset of district- and municipality-level characteristics, possibly associated with vulnerability to temperature. We identified factors that may explain geographical variation in vulnerability to extreme temperatures and subsequently operationalized them using data available at the district and municipality level. The resulting dataset included 21 variables on demographic, building, land-use, and weather characteristics. Data on demographic and building characteristics were available at the municipality and district level. Data on land-use and weather characteristics were assessed at the municipality and district level using administrative boundaries from 2019. More information on the meta-predictors, along with their definition and source, is provided in Table S1, https://links.lww.com/EE/A488.

The spatial distribution of the 21 meta-predictors displayed a highly fragmented spatial pattern (Figure S1, https://links.lww.com/EE/A488). The correlations between variables were comparable at the municipality and district levels (Figure S2, https://links.lww.com/EE/A488). To reduce the dimensionality of the meta-predictors dataset and ensure the predictive capabilities of the 21 variables, we performed a principal component analysis (PCA).^29^ This approach resulted in a new set of uncorrelated variables known as principal components (PCs). By reducing the number of indicators through the PCA, we were able to identify vulnerability patterns shaped by the combination of multiple small-area characteristics, avoiding arbitrary selections or computational challenges that might arise from handling a long list of variables.

### Statistical analysis

We assessed the association between short-term temperature exposure and all-cause mortality at the district level using a three-stage design. In the first stage, we applied the case time series design to estimate municipality-level temperature–mortality associations. The case time series design allows the application of flexible modeling techniques developed for time series analysis, without requiring the aggregation of the data into a single time series.^30,31^ Thus, for each of the 355 municipalities, we modeled the district-specific series of temperature and mortality using a conditional quasi-Poisson regression.^32^ We fitted the models separately for two age groups (0–74 and 75+). Temperature–mortality associations were modeled through distributed lag nonlinear models (DLNMs). DLNMs are a widely used framework to model immediate and delayed nonlinear exposure–response associations.^33^ Because the relationship between daily temperature and mortality shows lagged and nonlinear patterns, DLNMs are used extensively in the literature to estimate the temperature–mortality association.^34^ Within the DLNMs, risk associations are modeled through a cross-basis term, which defines the exposure–response and lag–response relationships. We modeled the exposure–response with a quadratic B-spline with three knots placed at the 10th, 75th, and 90th percentiles of the daily municipality-level temperature distribution. The lag–response was modeled with a natural cubic spline with three internal knots, placed at equally spaced distances on the lag log scale. The specification of the lag basis did not include an intercept. We defined a lag period of 0–21 days to capture delayed and indirect temperature effects on mortality. This parametrization of the exposure–and lag–response relationship is an established model specification from previous temperature–mortality studies.^34^ A term to account for within-district variations in baseline risks and temporal trends was included in the model. This term was defined by a district:year:month indicator. Additionally, the models were adjusted for a natural spline of time with 1 degree of freedom for each year included in the analysis to account for the remaining seasonal and long-term trends, and for a day-of-the-week indicator, in line with previous studies.^17,18^

In the second stage, we cumulated the overall risk over the lag dimension for each municipality and pooled the estimated coefficients for the two age groups and the 355 municipalities using a multivariate random-effects meta-regression.^35,36^ PCA indicators estimated at the municipality level were introduced into the model as meta-predictors to explain age group-specific between-municipalities variations in risk. The number of retained PCs was determined based on the proportion of variance explained, using a threshold of 80%. The contribution of the PCA indicators was evaluated using likelihood ratio tests, while the residual heterogeneity was assessed using the multivariate extension of Cochran’s Q test and the *I*^2^ statistic.

In the third stage, we employed the meta-regression model to predict the risks at the district level, using the PCA vulnerability indicators defined at this geographical level. We derived district-specific exposure–response curves, from which we extracted the minimum mortality temperature (MMT) and its related percentile (MMP). The MMT in each district was first defined as the temperature value for which the temperature-related mortality risk was the lowest. Because the district-specific MMTs showed considerable variability, including several extreme values, we decided to restrict the temperature range within which the MMTs could be defined. We restricted the interval to the country-level MMT (estimated by fitting the meta-regression model to the full population) plus or minus the standard deviation of the district-level MMT distribution (estimated with no interval restrictions) and bounded it within the 1st and 99th temperature percentiles. This adjustment narrowed the range between the 51st and 99th percentiles of the temperature distribution. We then computed the district-specific mortality relative risks (RR) and 95% confidence intervals (CIs) at the 99th and 1st temperature percentiles versus the MMT to obtain heat- and cold-related mortality RRs.

We also estimated age-specific yearly excess mortality, both as the total number of deaths and as a fraction of deaths, attributable to temperatures above (heat-related excess mortality) and below (cold-related excess mortality) the MMT, using a previously described method.^37^ These estimates were used to calculate age-specific rates of yearly heat- and cold-related excess mortality per 100,000 persons using as reference the 2019 population from Statistics Netherlands.^38^ Excess mortality rates were then standardized across age groups using age-specific standard weights.^39^ The related uncertainty was quantified as 95% empirical CIs using Monte Carlo simulations (n = 500). We tested differences in heat- and cold-related mortality in relation to four district-level variables: average income, percentage of tree cover, gas usage, and address density. For each variable, districts were divided into two groups—below and above the median value. We then examined whether total standardized mortality rates differed significantly between groups.

We conducted several sensitivity analyses. First, we modified the parametrization of the exposure–response function in the first-stage cross-basis term: in one model, the knot location was shifted to the 20th, 70th, and 85th temperature percentiles, while in another model, the quadratic B-spline was replaced with a natural spline. Second, we reduced the lag period to 3 days to evaluate the more direct effects of temperature. Finally, we repeated the analysis using temperature data from the ERA5 re-analysis dataset.

All statistical analyses were performed in R (version 4.5.0), using the *dlnm, gnm*, and *mixmeta* packages.^35,40,41^

## Results

The final analysis included 1,444,448 deaths from all causes in the period 2010–2019. On average, 869 deaths per 100,000 inhabitants occurred in each district in 2019 (Figure 1). Mean daily average temperature was characterized by a modest geographical gradient, ranging from 9.9°C in the north-eastern districts to 11.5°C in the southern and western districts (Figure 1). Minimum daily average temperature and maximum daily average temperature also showed geographical gradients, with districts along the coast displaying higher minimum temperatures and lower maximum temperatures compared with inland areas (Figure S3, https://links.lww.com/EE/A488). Mean, minimum, and maximum daily average temperatures by district estimated using the ERA5 re-analysis data are reported in the supplementary material (Figure S4, https://links.lww.com/EE/A488). The district-level daily temperatures estimated from the Bussalleu et al^26^ and ERA5 datasets were highly correlated. The mean Pearson correlation coefficient (standard deviation) of the district-specific correlations was equal to 0.992 (0.01).

**Figure 1.**
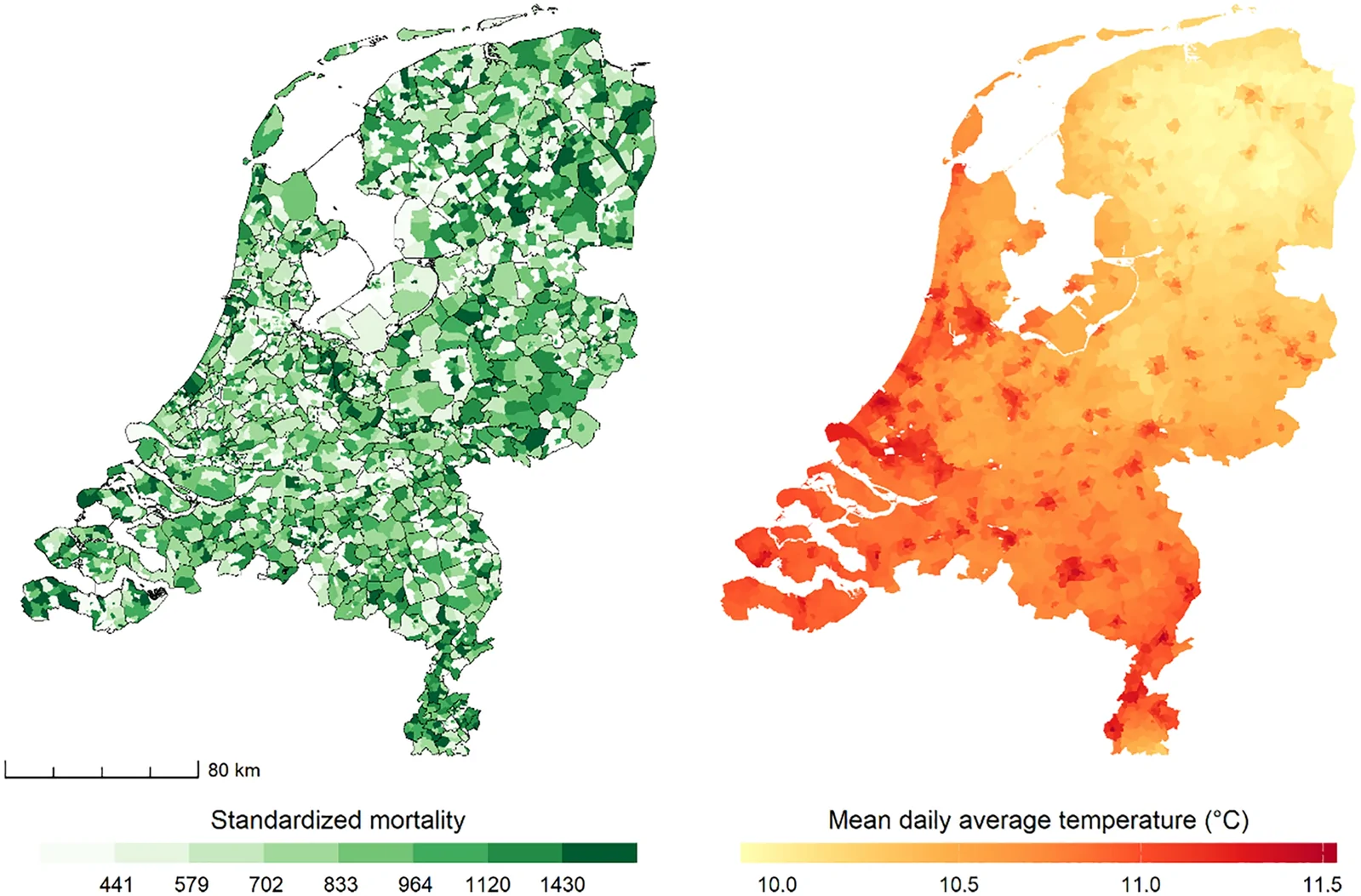
Standardized mortality × 100,000 inhabitants (left panel) in 2019 and mean daily average temperature (right panel) for the period 2010–2019, by district. Mortality counts were standardized using as reference the 2019 population from Statistics Netherlands.^26^

Figure 2 shows the pooled estimates of the overall cumulative exposure–response relationship by age group, predicted at the mean value of the meta-predictors. The pooled curve for the population aged 0–74 years showed moderate to null effects of temperature on mortality. Especially at the temperature extremes, the curve showed no increase in mortality risk. The curve for the 75+ age group displayed the usual V-shape from the 5th temperature percentile. However, from the minimum temperature until the 5th percentile, the RR of mortality decreased with decreasing temperatures. The MMT for both age groups was relatively high, corresponding to the 99th and 94th percentile for the younger and older age groups, respectively (Table S2, https://links.lww.com/EE/A488). The heat-related and cold-related RRs [95% CI] for the younger population were equal to 1.00 [1.00–1.00] and 1.08 [0.99–1.18], at the 99th and 1st temperature percentiles (Table S2, https://links.lww.com/EE/A488). The RRs for the population aged 75+ were equal to 1.05 [1.01–1.11] and 1.18 [1.11–1.25] at the 99th and 1st percentiles, respectively (Table S2, https://links.lww.com/EE/A488).

**Figure 2.**
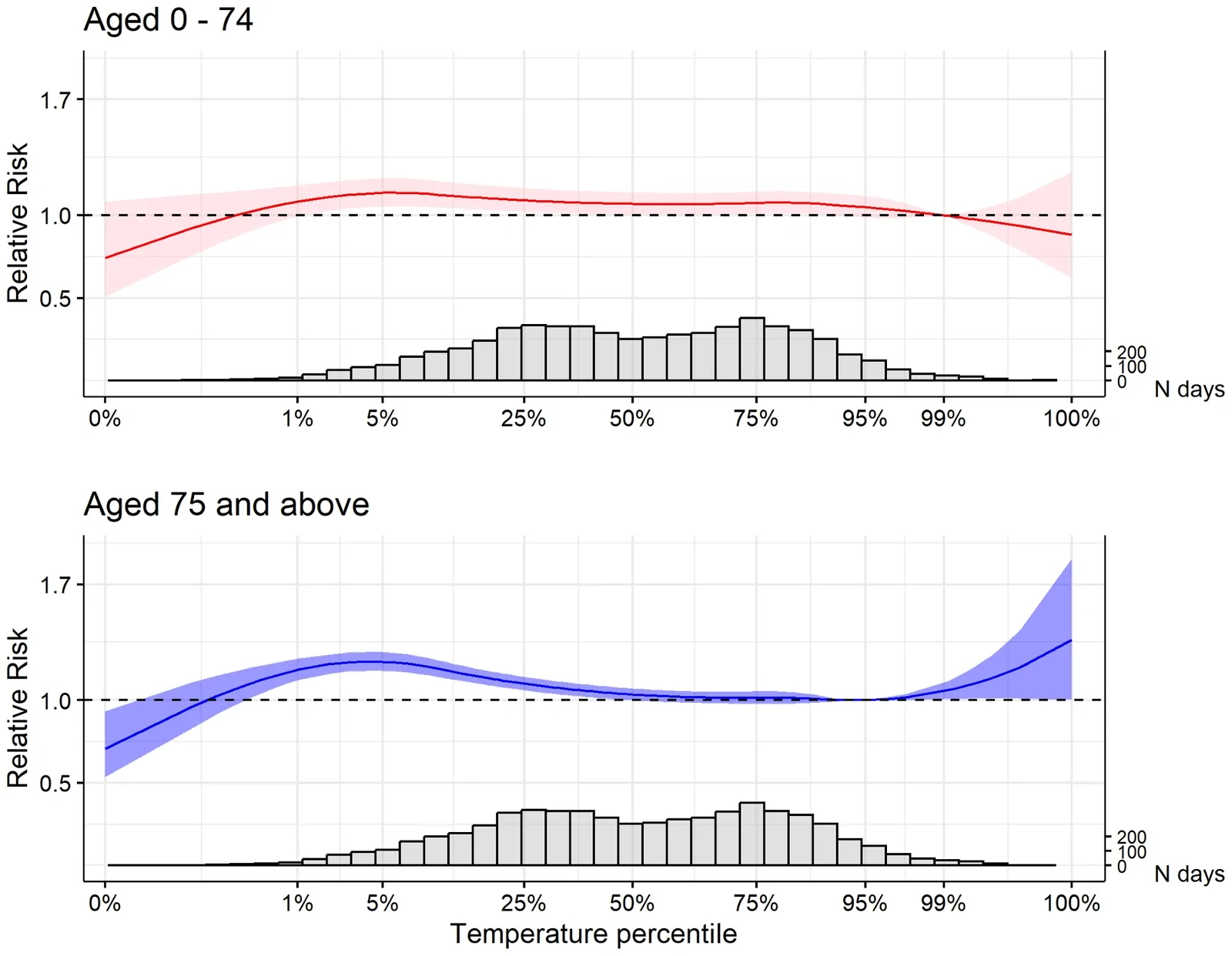
Pooled estimates of the overall cumulative exposure–response relationships between temperature percentiles and all-cause mortality for two age groups (0–74; 75+). The histograms display the daily average temperature distribution across all districts (N days = 3,652).

We included five PCs as meta-regressors in the second-stage model, which explained 78% of the variance (Figure S5, https://links.lww.com/EE/A488). The geographical distribution of the five PCs and the correlations between the PCs and the 21 variables are presented in Figures S6 and S7, https://links.lww.com/EE/A488. Despite the inclusion of the meta-predictors, the meta-regression model still showed some residual heterogeneity (Cochran’s Q test: *P* = 0.01; *I*^2^ = 5.0%), indicating that a proportion of the variability in risk across municipalities was not explained by the model. This finding was supported by the likelihood ratio test, which identified only age and PC1 as statistically significant in the meta-regression model (Table S3, https://links.lww.com/EE/A488).

Figure 3 shows the geographical distribution of the yearly standardized excess mortality rates attributable to heat and cold across districts and municipalities. Heat- and cold-related excess mortality rates displayed different, yet not strictly opposite, patterns. District-level maps displayed high heat-related excess mortality rates, especially in densely populated and urbanized districts. Cold-related mortality rates were larger in magnitude than heat-related rates; they displayed moderate spatial gradients, with higher vulnerability observed in districts located in the North, South-West, and South-East regions, compared to districts located in the center of the country (Figure 3). Compared with the district maps, the municipality maps showed similar yet more coherent and interpretable spatial patterns of excess mortality rates (Figure 3).

**Figure 3.**
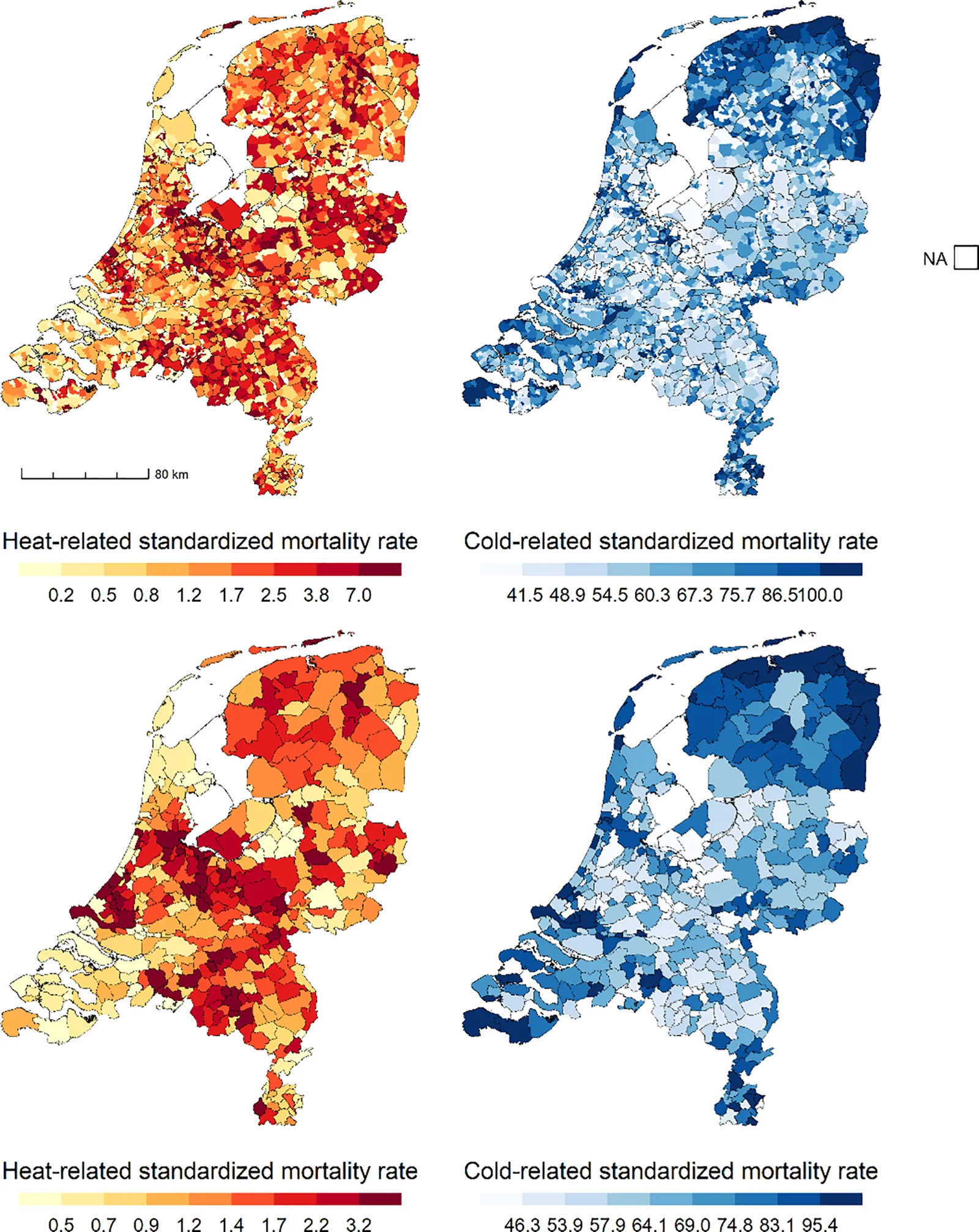
Geographical distribution of yearly standardized excess mortality rates (× 100,000 person-years) attributable to heat and cold across districts (top row) and municipalities (bottom row) in the Netherlands. The legend intervals are not equally spaced, but correspond to the 10^th^ percentiles of the distribution of the standardized mortality rates. Mortality rates were set to NA for districts with less than 10 deaths across the whole study period (N districts = 183).

Additional district-level effect summaries stratified by age group are presented in the supplementary material (Table S4, https://links.lww.com/EE/A488). The MMT exhibited considerable variation across districts, ranging from 11°C to 25°C (Figure S8, https://links.lww.com/EE/A488). Geographic gradients were evident in both MMTs and MMPs, with lower values generally observed in inland and urban areas. Figure S9, https://links.lww.com/EE/A488 presents the geographical distribution of the RRs at the 99th and 1st temperature percentile vs. the MMT. Among individuals aged 0–74 years, heat-related RRs were generally close to 1, with few exceptions; cold-related RRs were higher in magnitude and displayed greater variability. The RRs for the 75+ population were consistently higher than the ones estimated for the younger age group, and displayed marked spatial variability. Similarly, to the standardized mortality rates, the distribution of the RRs in the older age group suggested increased susceptibility to heat in more urbanized areas.

Table S5, https://links.lww.com/EE/A488, shows the annual excess deaths and the standardized excess mortality rate per 100,000 person-years for the whole country. On average, across the study period, temperatures above the MMT were associated with 346 excess deaths (empirical 95% CI = 44, 558) each year, and those below the MMT with 11,988 excess deaths (7,554–15,609) each year, corresponding to a standardized excess mortality rate of 2.2 deaths (empirical 95% CI = 0.3, 3.5) per 100,000 person-years for heat, and 73.3 deaths (46.8–94.7) per 100,000 person-years for cold.

Figure 4 displays the standardized excess mortality rate per 100,000 person-years, stratified by district-level average income, percentage of tree cover, gas usage, and address density. Weak differences in standardized mortality rates were observed for districts with different income levels, with higher heat-related mortality observed in higher-income districts and higher cold-related mortality in lower-income districts. Although the district and municipality-level maps indicate high heat-related standardized mortality rates in urbanized areas, no significant differences in standardized mortality rates were found between districts with low and high address density.

**Figure 4.**
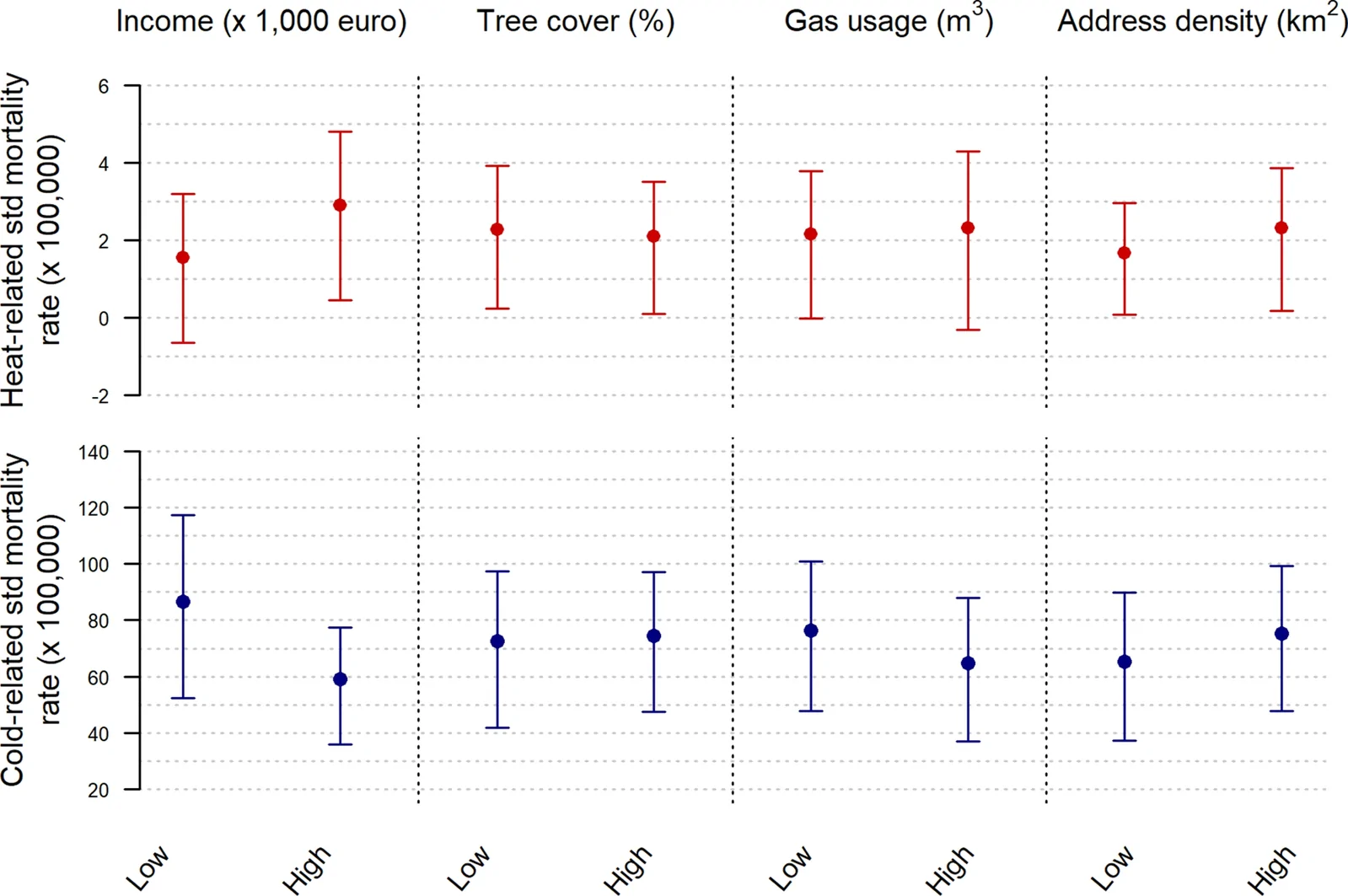
Standardized excess mortality rates (× 100,000 person-years) attributable to heat and cold in the Netherlands, stratified by district-level tree cover, income, gas usage, and address density.

Changing the position of the knots in the exposure–response parametrization and using temperature data with coarser spatial resolution (from ERA5) did not alter the main findings (Figure S10, https://links.lww.com/EE/A488). Exposure–response curves modeled through a natural spline displayed no decrease in the RR of mortality at extremely cold temperatures for both age groups, in contrast to the curves modeled with a B-spline. Applying a lag period of 3 days also influenced the shape of the exposure–response curves, with increased heat-related risk in both the young and elderly population and no risk associated with cold temperatures (Figure S10, https://links.lww.com/EE/A488).

## Discussion

This study provides a country-wide assessment of temperature-mortality associations across 3,116 districts in the Netherlands between 2010 and 2019. Our maps indicate that heat-related excess mortality was elevated in urban areas, and cold-related excess mortality was elevated in the northern and southern regions. Increased heat- and cold-related mortality risks were observed for the elderly (75+ years of age), while for the younger population (0–74 years of age) null to moderate associations were reported. Mortality risks were moderate in mild cold, while extreme cold was associated with lower mortality. We found a weak indication of higher heat-related standardized excess mortality in high-income districts and of higher cold-related standardized excess mortality in low-income districts. No differences in standardized mortality rates were found in relation to district-level percentage of tree cover, gas usage, and address density. During the study period, heat (i.e., ambient temperatures above MMT) was associated with an average of 346 deaths per year, and cold with 11,988 deaths per year. This corresponds to a heat-related standardized excess mortality rate of 2.2 [0.3–3.5] per 100,000 person-years, and a cold-related rate of 73.3 [46.83–94.74] per 100,000 person-years.

District-level maps of temperature-related standardized mortality rates displayed pronounced spatial heterogeneity in heat-related mortality. Such spatial variability might have been caused not only by differences in location-specific socio-physiological vulnerabilities to temperature, but also by methodological factors. In particular, the estimated district-level MMTs determined the number of days classified as a heat or cold day, depending on where the MMT fell within each district’s temperature distribution. Districts with higher MMTs experienced fewer days classified as heat, and thus, by definition, exhibited lower heat-related excess mortality rates compared to districts with lower MMTs. Aggregating the effect summaries at the municipality level revealed more coherent spatial patterns in heat-related mortality, with increased mortality rates observed in densely populated municipalities. High heat-related mortality was also found predominantly in urbanized areas in England and Wales,^17^ and in France;^19^ contrarily, rural areas displayed higher heat-related mortality rates compared to urban areas in Norway.^18^ Our maps displayed higher cold-related mortality rates in the North, South-West, and South-East regions of the Netherlands. Similarly, in England and Wales, elevated cold-related mortality was observed in more remote areas (northern England and south-western regions);^17^ in Norway and France, cold-related mortality did not exhibit clear geographical clustering.^18,19^

Our results align with previous research indicating a significant effect of age on temperature-related mortality. While moderate to null effects were found in the 0–74 age group, the 75+ age group showed high heat- and cold-related mortality risks at the temperature extremes, with the exposure–response curve exhibiting the characteristic V-shape after the 5^th^ temperature percentile. Until the 5th percentile, the exposure–response function indicated a decreasing risk of mortality. This apparent anomaly may reflect the difference between outdoor temperatures and actual population exposure, as individuals are more likely to stay indoors during extreme cold temperatures.^18,34,42^ We found a weak indication of higher heat-related standardized excess mortality in high-income districts and of higher cold-related standardized excess mortality in low-income districts. Similarly, to our results, other studies found increased cold-related mortality rates in neighborhoods with greater socioeconomic deprivation and lower educational attainment.^17,18^

We found no differences in standardized mortality rates in relation to district-level percentage of tree cover, gas usage, and address density. In line with our results, two other small-area studies did not find significant effect modification by a wide range of contextual factors.^12,19^ Contrarily, few studies have reported evidence of significant effect modification by sociodemographic and environmental factors. For example, increased heat-mortality rates have been observed in urban compared to rural areas, and in municipalities characterized by lower educational attainment.^12,17,18^ One possible explanation for the lack of significant differences in excess mortality rates in relation to contextual factors in our analysis is that, in our study area, vulnerability to temperature is better described by a combination of vulnerability factors, and not by single area-level characteristics. This explanation is supported by de Schrijver et al,^12^ who found that in rural areas, population aging and socioeconomic factors appear to play a more significant role in modifying heat-related risks, while the main drivers for heat-related mortality in urban areas were environmental factors, such as temperature and air pollution. Another explanation is that the use of district-level variables may mask important differences present at the individual level. Thus, our findings do not rule out the possibility of effect modification at the individual level. In addition, by testing differences in mortality rates only between two groups (above and below the median), we might have missed differences among groups with greater contrast.

In line with global findings, our study found substantially greater effects of cold compared to heat on mortality.^1,34^ This result is particularly important as epidemiological projections estimate that under different climate scenarios of global warming, cold-related mortality is expected to stay constant or even increase in the Netherlands due to population aging.^43^ At the same time, a few factors must be considered when interpreting the cold-related mortality burden estimated in the current analysis. First, the association between cold temperatures and mortality appeared to be sensitive to the choice of lag period. Specifically, when a 3-day lag was applied, the cold-related RRs largely disappeared, suggesting that cold-related mortality may be driven mostly by indirect and delayed effects of cold exposure, rather than its direct impacts. In addition, the attribution of heat- and cold-related mortality was largely determined by the position of the reference temperature (MMT) within the overall temperature distribution. In our study, the country-level MMT (MMP) for the younger and older age groups were equal to 23.4 (99th) and 19.7 (94th), respectively. Consequently, the majority of days throughout the study period contributed to cold-related excess mortality, whereas heat effects were restricted to the relatively few days with temperatures above the MMT. Comparably high MMPs were also observed in Norway (93rd and 94th percentiles for females and males),^18^ and in England and Wales (95th percentile).^17^ In contrast, earlier Dutch studies using aggregated temperature data reported lower MMPs and corresponding MMTs. The study by Huynen et al^21^ found an MMT of 16.5°C, and the study by Folkerts et al^23^ found an MMT of 15.3°C in the 65+ age group. The observed higher MMTs compared with previous studies may be attributable to methodological differences, such as the use of modeled district-level temperature data, as opposed to temperature measurements from monitoring stations used in earlier research.

Our findings are also consistent with recent European studies employing similar methodologies to assess country-level small-area mortality risks.^17,18^ These studies reported heat-related excess mortality rates of 1.6 [1.2–1.9] and 0.8 [0.6–1.1] per 100,000 person-years in England and Wales, and Norway, respectively, and cold-related excess rates of 122.3 [112.9–131.5] and 73.4 [69.7–77.1] per 100,000 person-years.^17,18^ Multicountry and multicity (MMC) studies have also yielded comparable mortality estimates across European countries. For instance, the study by Masselot et al^44^ reported a heat-related standardized mortality rate of 9 [7–10] and a cold-related rate of 83 [74–92] deaths per 100,000 person-years across 854 urban areas in Europe during the years 2000–2019. The heat-related standardized rates from the MMC studies are on average larger than the rates found in our study. This is partly due to differences in the position of the reference temperature. As mentioned above, studies conducted using aggregated data have reported lower MMTs compared to our study, which likely results in higher heat-related mortality rates.

Notably, the sensitivity analysis using coarser-resolution temperature data produced estimates comparable to those obtained in the main analysis (Figure S10, https://links.lww.com/EE/A488). This result is particularly noteworthy, as it indicates that coarser temperature data may adequately characterize temperature-related risk without requiring highly detailed temperature spatial information. Similar findings were reported by Mistry et al,^45^ who showed that temperature-related risk estimates derived from ERA5 re-analysis data were comparable to those based on station observations.

### Strengths and limitations

A key strength of this study is the use of high-resolution temperature data aggregated at a small-area level. This approach overcomes the limitations of analyses based on temperature data aggregated over large geographical areas, which fail to capture fine-scale variation in temperature-mortality associations. In addition, the use of well-established methodological frameworks allowed us to derive multiple effect estimates, facilitating direct comparison of our results across Dutch regions and with other European studies. Another methodological strength is the use of PCs as meta-predictors in the meta-regression model instead of individual vulnerability variables. This approach enabled correlated local characteristics to be summarized into a smaller number of composite predictors, reducing the dimensionality of the dataset and minimizing collinearity between variables.

Despite these strengths, several limitations should be acknowledged. First, the parametrization of the exposure–response curve possibly introduced uncertainty in the estimation of heat-related RRs. Specifically, because most district-specific MMPs were above the 90th percentile, the placement of knots at the 10th, 75th, and 90th percentiles may have affected the model’s ability to capture heat-related RRs. Thus, there was greater uncertainty associated with estimated heat-related risks and, consequently, with heat-related excess mortality rates. Second, analyses were conducted at the municipality level and subsequently downscaled to districts, with the assumption that baseline mortality risk was homogeneous across districts within the same municipality, and that between-district differences were primarily driven by meta-predictor values. While this assumption simplifies the modeling framework and facilitates meaningful geographic clustering—useful for informing public health interventions—it may have smoothed out local variabilities in risk. Third, the use of time-fixed district-level meta-predictors limited our ability to examine the influence of evolving local characteristics on the mortality risks over the study period. A fourth limitation of this study is that we estimated associations for only two broad age groups. As a result, differences in vulnerability between narrower age categories may have been overlooked. However, assessing the temperature-mortality associations for more and narrower age groups would have resulted in a statistical power issue, especially for younger populations.

Finally, two considerations should be made about the use of PCs as meta-predictors in the meta-regression model. First, although we incorporated five PCs as meta-predictors, explaining 78% of the variance in the area-level characteristics, the meta-regression still showed moderate residual heterogeneity between municipalities. This indicates that the set of PCs and underlying area-level variables included in our model did not fully account for the variation in baseline risk across municipalities. Second, while the PCA was effective in reducing data complexity, it precluded assessment of the independent effects of specific vulnerability factors. However, the absence of significant differences in mortality rates across districts with different levels of tree cover, gas usage, and address density may suggest that, at least in the Netherlands, composite indicators may better capture the multifactorial nature of vulnerability to temperature exposure compared to single variables.

## Conclusion

District-level maps of temperature-related standardized mortality rates showed that elevated heat-related mortality rates were more commonly observed in urbanized districts, and that cold-related mortality was higher in the North, South-West, and South-East regions of the country. The 75+ group showed more pronounced mortality risks compared to the younger age group. A small difference in heat- and cold-related standardized mortality rates was found between districts with low and high income, whereas no differences were observed between districts with different levels of tree cover, gas usage, or address density. Importantly, the use of small-scale data enabled risk estimation at a fine spatial resolution, revealing local heterogeneity that may be overlooked in analyses at broader geographical scales. These findings may help to inform public health policy in the Netherlands and support future assessments of temperature-related health risks under climate change.

## ACKNOWLEDGMENTS

The authors thank Danny Houthuijs, Frank den Hertog, Cor Jacobs, Guus Velders, Lisbeth Hall, Fons van der Lucht and Ronald Gijsen for their contribution to the conceptualization of the study.

## Supplementary Material
