# Supplementary material for: District-level analysis of temperature-related excess mortality in the Netherlands
Source: Environ Epidemiol. 2026 Oct 15;10(6):e534. doi: 10.1097/EE9.0000000000000534 (PMC13674960; doi:10.1097/EE9.0000000000000534)
Supplement: Supplementary file 1 [file ee9-10-e534-s001.pdf]

Supplementary Information of

District-level analysis of temperature-related excess mortality in the Netherlands

Aisha Ndiaye[a], Hein Daanen[b], Jan van de Kassteele[a], Kees de Hoogh[c], Laurens Zwakhals[a], Liselotte van Asten[a], Werner Hagens[a], Jochem Klompmaker[a]

[a] National Institute for Public Health and the Environment (RIVM); Bilthoven, the Netherlands
[b] Department of Human Movement Sciences, Faculty of Behavioural and Movement Sciences, Vrije Universiteit Amsterdam; Amsterdam, The Netherlands
[c] Institute for Risk Assessment Sciences (IRAS), Utrecht University; Utrecht, the Netherlands

Corresponding author:
Aisha Ndiaye
aisha.ndiaye@rivm.nl
+31 652904173
Antonie van Leeuwenhoeklaan 9, 3721 MA
Bilthoven, the Netherlands

Table S1. List and definition of the 21 variables included in the principal component analysis (PCA).

Variable	Definition	Year	Source
**Demographic**			
Pop above 75 (%)	Percentage of people aged above 75 years	2019	CBS(1)
Low education (%)<br><br>High education (%)	Percentage of persons who were between 15 and 75 years old on October 1 and were registered in a Dutch municipality, of whom the highest attained level of education is low or high education. Low education includes education at the level of primary education, vmbo, the first 3 years of havo/vwo, the former assistant training (mbo1), and vocational education.[1] Higher education includes education at the level of higher professional education or university.	2019	CBS
Income (x 1,000 euro)	Average personal income based on individuals with personal income who are part of private households	2019	CBS
Widowed (%)	Percentage of inhabitants who were widowed on January 1. The marital status widowed arises after the dissolution of a marriage or registered partnership due to the death of the partner	2019	CBS
One person household (%)	Percentage of one person households (i.e. private household consisting of one person)	2019	CBS
Unemployed (%)	Percentage of people receiving unemployment benefit under the Unemployment Insurance Act (WW)	2019	CBS
Disability (%)	Percentage of people incapacitated to work (i.e. persons receiving disability benefits under the Disability Insurance Act (WAO), the Self-Employed Disability Insurance Act (WAZ), the Act on Work and Labor Support for Young Handicapped Persons (Wajong), and the Work and Income According to Labor Capacity Act (WIA))	2019	CBS

[1] Vmbo: pre-vocational education; havo: higher general continued education; vwo: preparatory scientific education.

Address density (km^2)	Average number of addresses per square kilometer within a circle with a radius of one kilometer on January 1st 2019	2019	CBS
**Building**			
Dwellings built before 2000 (%)	Percentage of houses built before 2000 expressed as whole percentages of the total number of homes	2019	CBS
One family dwelling (%)<br><br>More than one family dwelling (%)	Percentage of “eengezinswoning” and “meergezinswoning”. (Eengezinswoning: Any dwelling that also constitutes an entire building. This includes detached houses, attached houses, such as semi-detached houses, farms with residences, and all terraced houses;<br>Meergezinswoning: Each dwelling that, together with other residential or commercial spaces, forms a whole building. This includes flats, gallery houses, porch houses, ground- and upper-floor apartments, and residences above commercial spaces, provided they have an entrance door located outside the commercial space)	2019	CBS
Electricity usage (kWh)<br><br>Gas usage (m^3)	Average electricity and gas consumption for all types of housing combined	2019	CBS
Rented dwelling (%)	Percentage of homes that are not occupied by the owner of the property. In cases where no resident is registered, it concerns homes that are likely intended for the rental market	2019	CBS
**Land-use**			
Tree cover (%)	Percentage of land covered by trees > 2.5m. All trees taller than 2.5 meters are represented in a grid map with a resolution of 10x10 meters. The trees are expressed as a percentage of trees per grid cell	2022	Atlas Natuurlijk Kapitaal
Green cover (%)	Percentage of land area covered by green areas. All trees, bushes, and low vegetation are represented in a grid map with a resolution of 10x10 meters. The green areas are	2022	Atlas Natuurlijk Kapitaal

	expressed as a percentage of trees per grid cell		
Water cover (%)	Percentage of land area covered by water	2023	Kilmaat Effect Atlas
Distance from coast (m)	Shortest distance between coast and wijk and geemente centroids	2019	Calculated in R
**Weather**			
Max temp summer (°C)<br><br>Min temp winter (°C)	Maximum temperature during summer season;<br>Minimum temperature during winter season	2019	Utrecht University(2)

Figure S1. Geographical distribution of the 21 small-area variables across the 3,116 districts in the Netherlands.

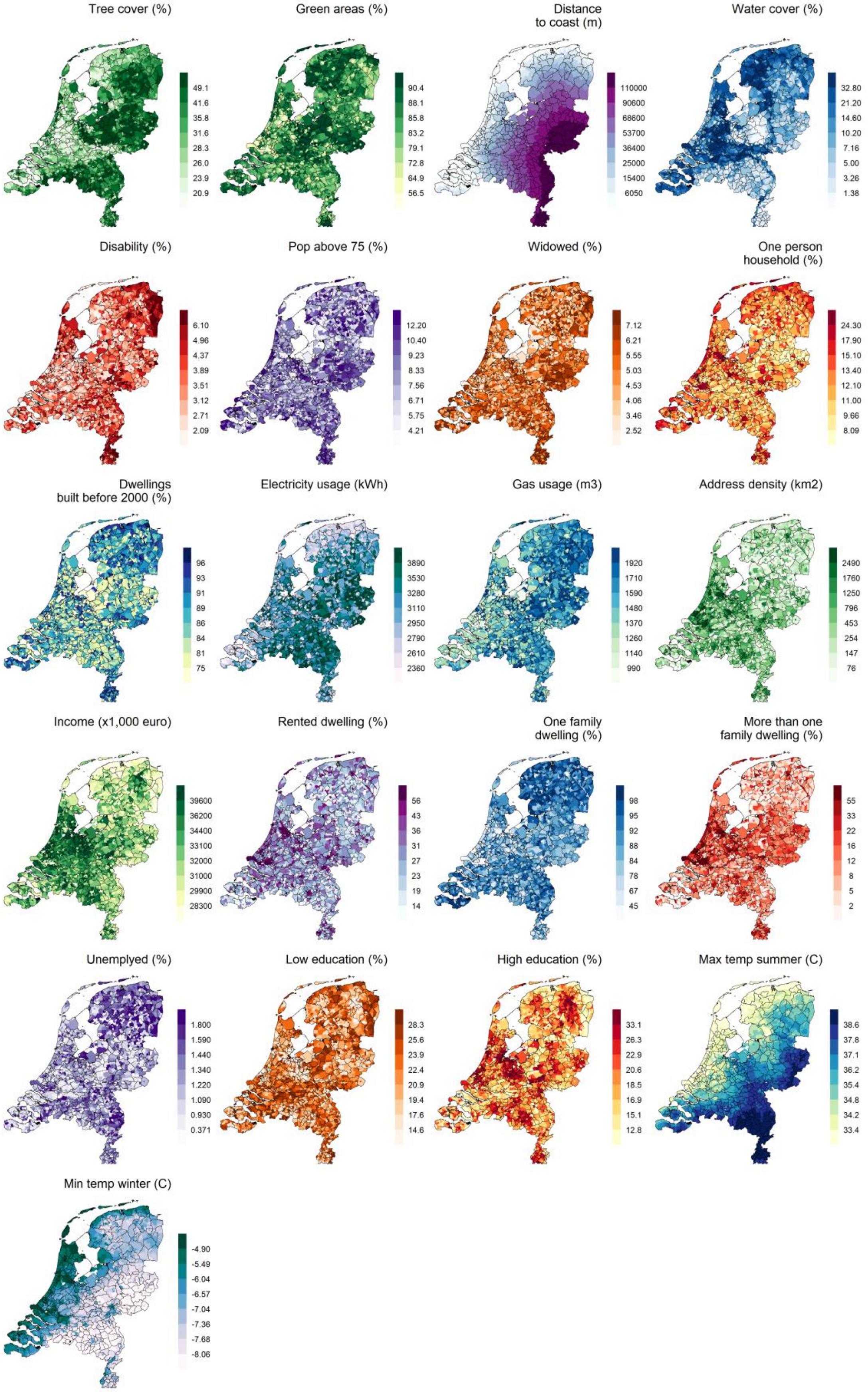

Figure S2. Pearson correlation plots of the 21 small-area variables at the district level (bottom-left triangle) and municipality level (top-right triangle).

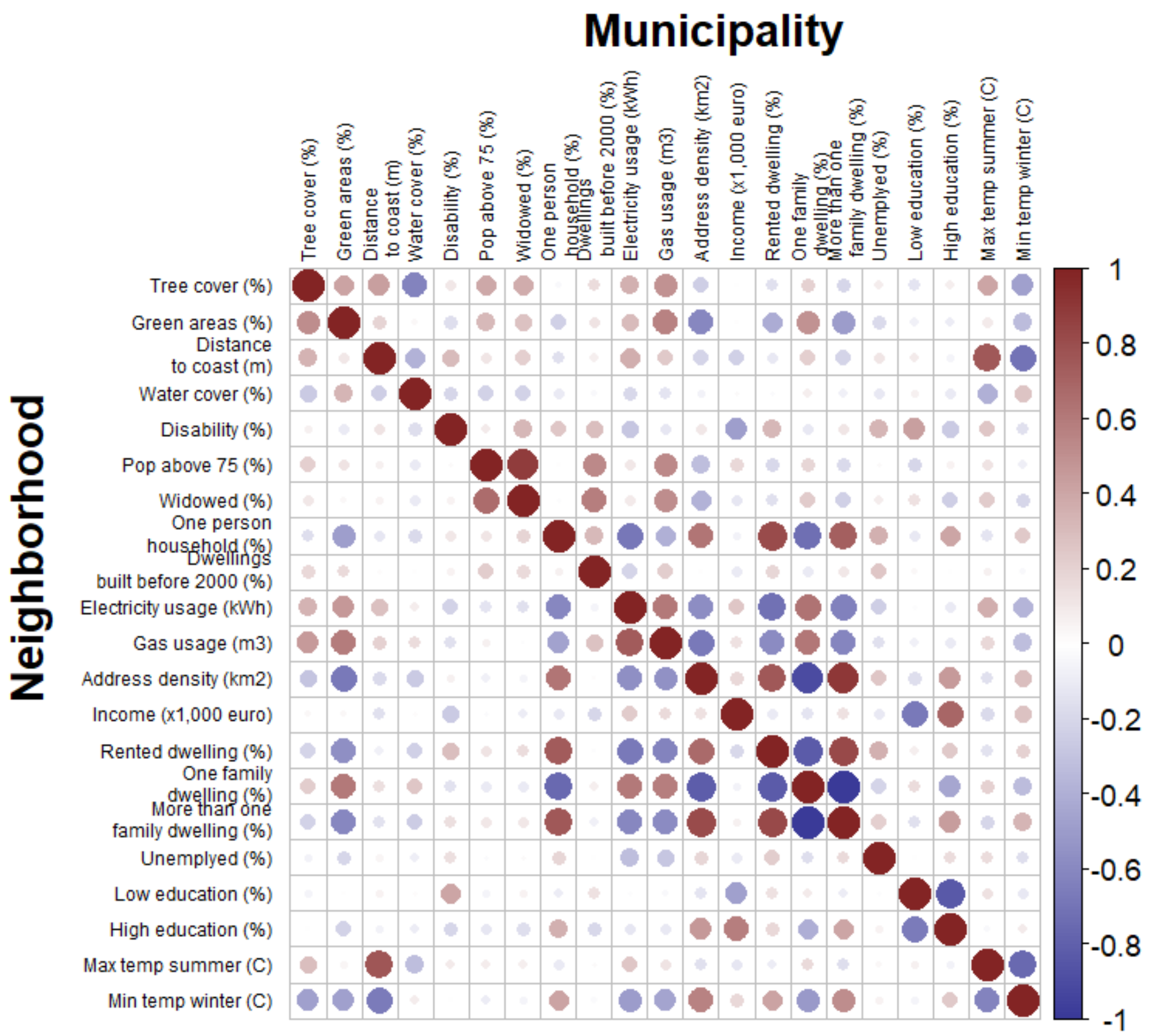

Figure S3. Minimum (left panel) and maximum (right panel) daily average temperature by district in the Netherlands for the years 2010-2019, calculated using modelled daily ambient temperature at a 1 x 1 km scale.

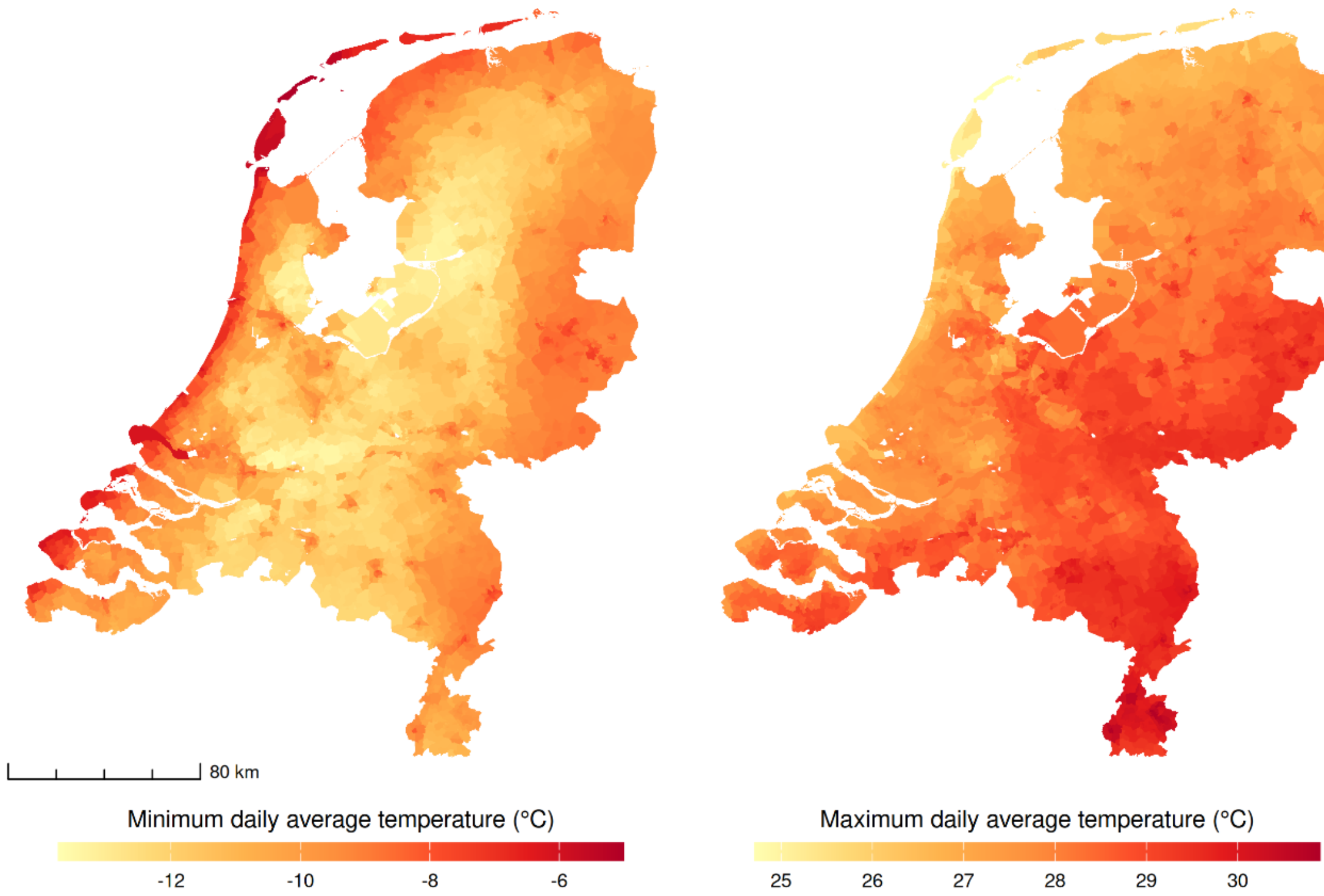

Figure S4. Mean, minimum and maximum daily average temperature by district in the Netherlands for the years 2010-2019, calculated using ERA5 re-analysis data at a 28 x 28 km scale.

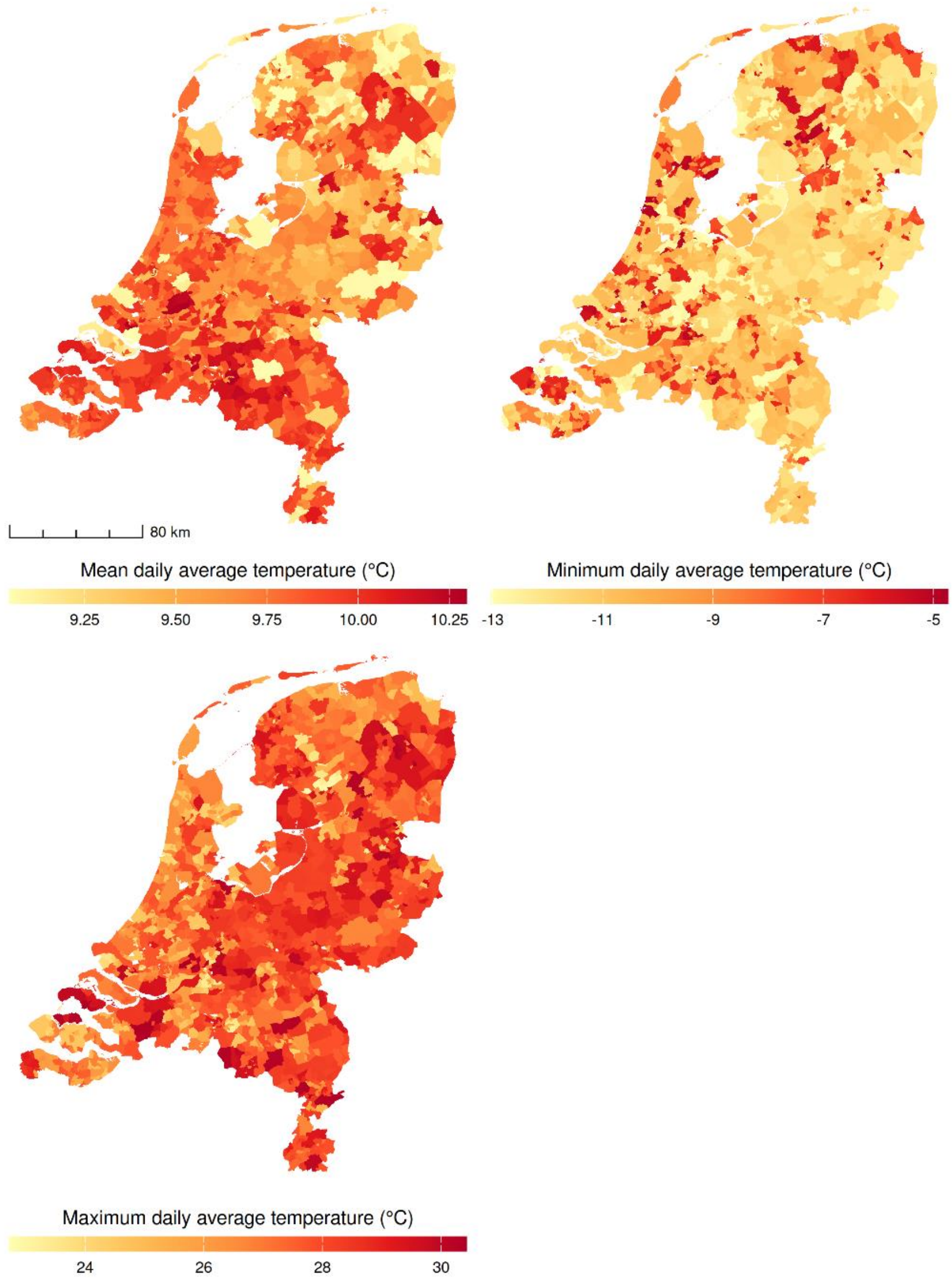

Table S2. Summary statistics of the pooled exposure response-curves, for the two age groups (0-74; 75+).
MMT = minimum mortality temperature (°C).
MMP = minimum mortality temperature percentile (%).
T (°C) = temperature in degrees Celsius.
RR = relative risk at the 1st and 99th temperature percentile vs. the MMT.

**Age group**	**MMT**	**MMP**	**T (°C) 1st percentile**	**RR (95% CI) 1st percentile**	**T (°C) 99st percentile**	**RR (95% CI) 99th percentile**
0-74	23.4	99	-3.2	1.08 [0.99-1.18]	23.4	1.00 [1.00-1.00]
75+	19.7	94	-3.2	1.18 [1.11-1.25]	23.4	1.05 [1.01-1.11]

Figure S5. Cumulative variance plot of the principal component analysis (PCA) of the 21 small-area variables at the municipality level.

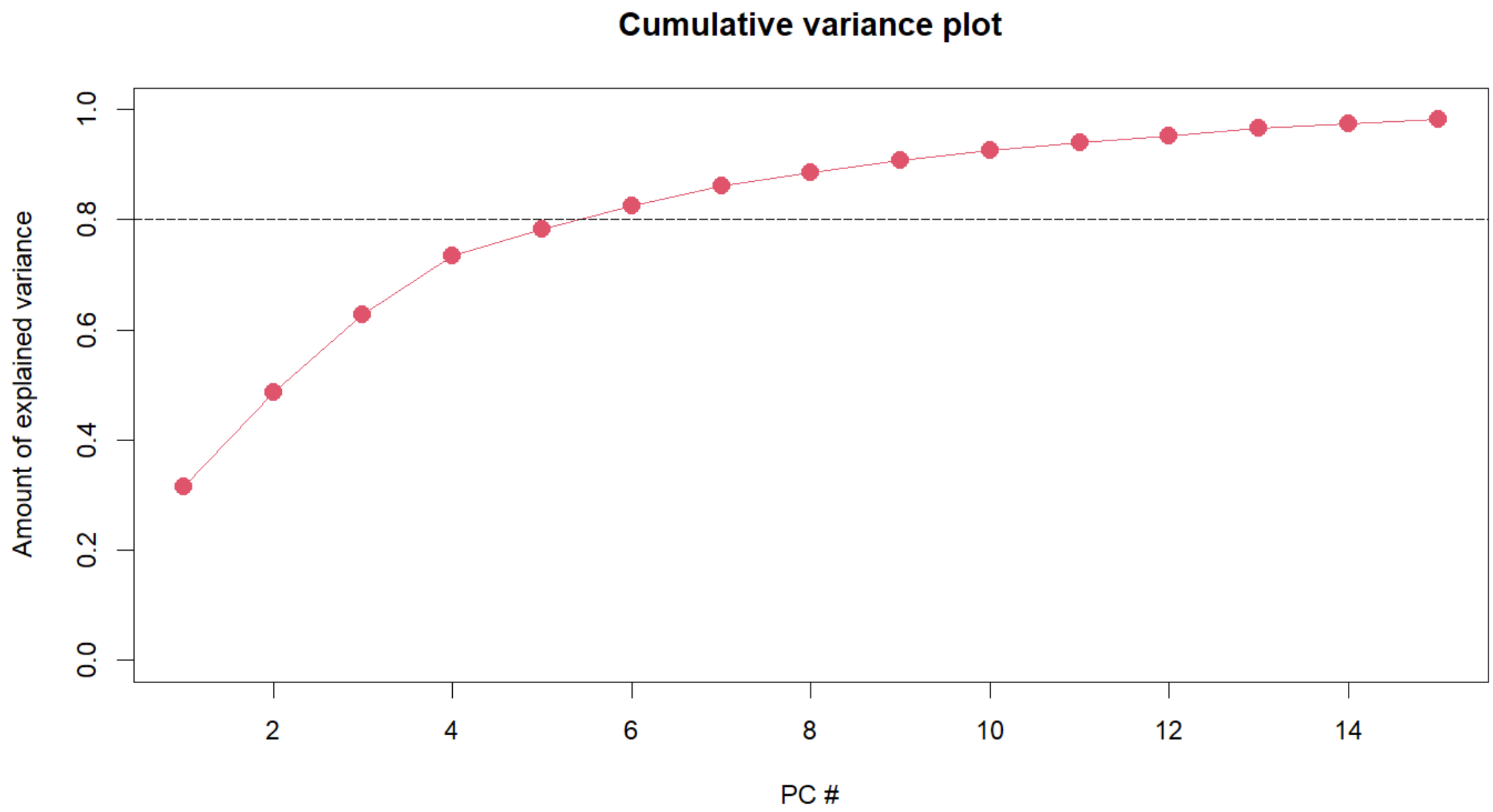

Figure S6. Geographical distribution of the first five principal components used as composite meta-predictors across the 3,116 districts in the Netherlands.

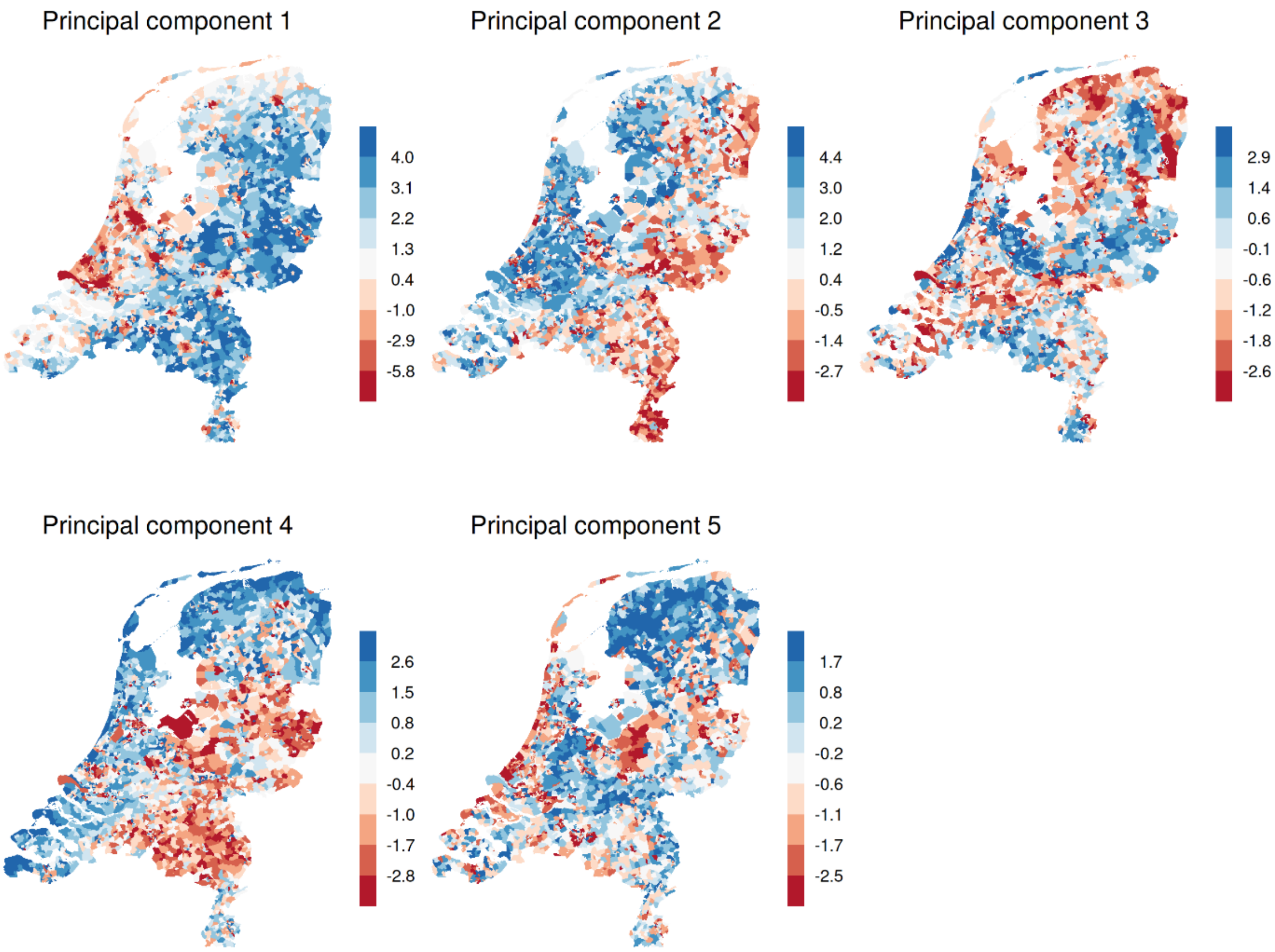

Figure S7. Heatmap representing the correlation between the first six principal components (y-axis) and the original 21 small-area variables (x-axis). The first five (highlighted) components capture most of the correlations (PCA scores) with the original variables and were used as predictors in the meta-regression.

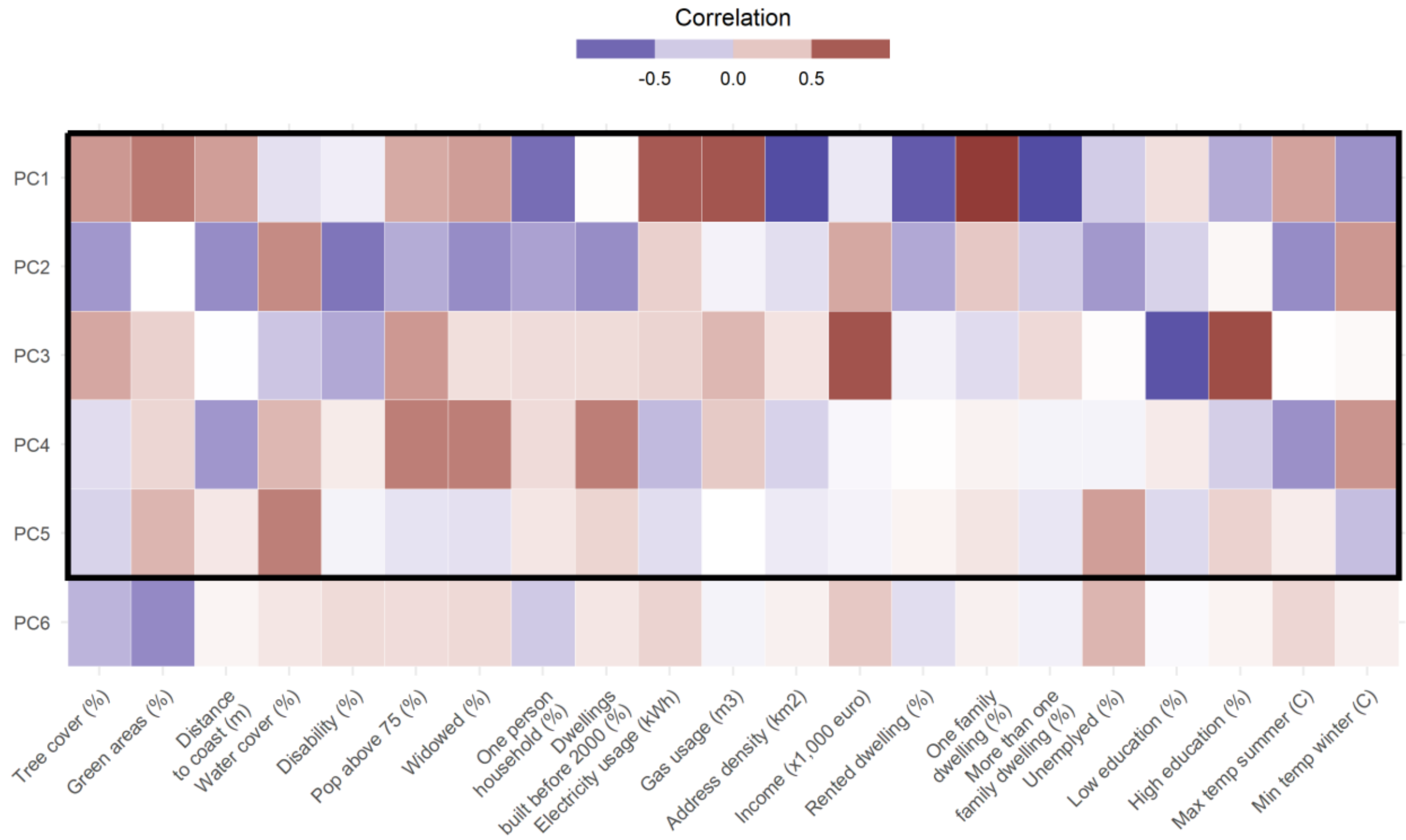

Table S3. Summary of the likelihood ratio test used to test the contribution of each predictor in the second-stage meta-analytical model. The AIC score is expressed for the sequence of models with an increasing number of predictors. The p-value indicates whether dropping a specific predictor from the main model significantly worsens the model fit.
AIC = Akaike Information Criterion

**Model**	**AIC**	**p-value**
Age	12092	0.00
PC1	12066	0.02
PC2	12062	0.09
PC3	12059	0.29
PC4	12058	0.39
PC5	12056	0.75

Table S4. Summary statistics by district in the Netherlands for the years 2010-2019.
RR = relative risk at the 1st and 99th temperature percentile vs. the MMT.
AF = attributable excess mortality fraction (%).
MMP = minimum mortality temperature percentile (%).
MMT = minimum mortality temperature (°C).
Rate = standardized excess mortality rate (x 100,000 person-years) for temperatures below and above the MMT.

**Summary statistic**	**effect**	**Age group**	**Mean**	**Min**	**25th**	**Median**	**75th**	**Max**
RR	cold	0-74	1.09	0.55	0.99	1.06	1.15	3.15
		75+	1.19	0.62	1.08	1.15	1.26	3.49
	heat	0-74	1.01	1.00	1.00	1.00	1.00	1.28
		75+	1.07	1.00	1.03	1.06	1.10	1.41
AF	cold	0-74	8.78	-9.16[2]	6.51	8.51	10.56	31.90
		75+	7.35	-27.63	5.34	6.97	9.20	30.78
		all	7.94	-6.66	6.15	7.57	9.44	31.04
	heat	0-74	0.09	-2.53	-0.07	-0.02	0.01	10.66
		75+	0.48	-2.35	0.11	0.24	0.59	10.02
		all	0.31	-2.10	0.06	0.14	0.35	6.58
MMP	-	0-74	95.9	51.0	99.0	99.0	99.0	99.0
		75+	83.3	52.0	70.0	92.0	96.0	99.0
MMT	-	0-74	22.3	11.0	22.0	23.1	23.9	25.7
		75+	17.7	11.0	15.0	19.0	20.0	25.0
Rate	cold	-	65.24	-81.61[1]	48.01	61.84	81.98	288.46
	heat	-	2.74	-9.87	0.53	1.36	3.29	45.62

[2] Negative values for the AF and mortality rate in some neighborhoods occur because these metrics are calculated based on the relative risk (RR). When the RR curve falls below one (i.e., RR < 1.00), both the attributable fraction and rate become negative.

Figure S8. Geographical distribution of the minimum mortality temperature (MMT, in °C - left) and its related percentile (MMP, in % - right) across districts in the Netherlands. The maps display the average district-level MMT and MMP, calculated across both age groups.[3]

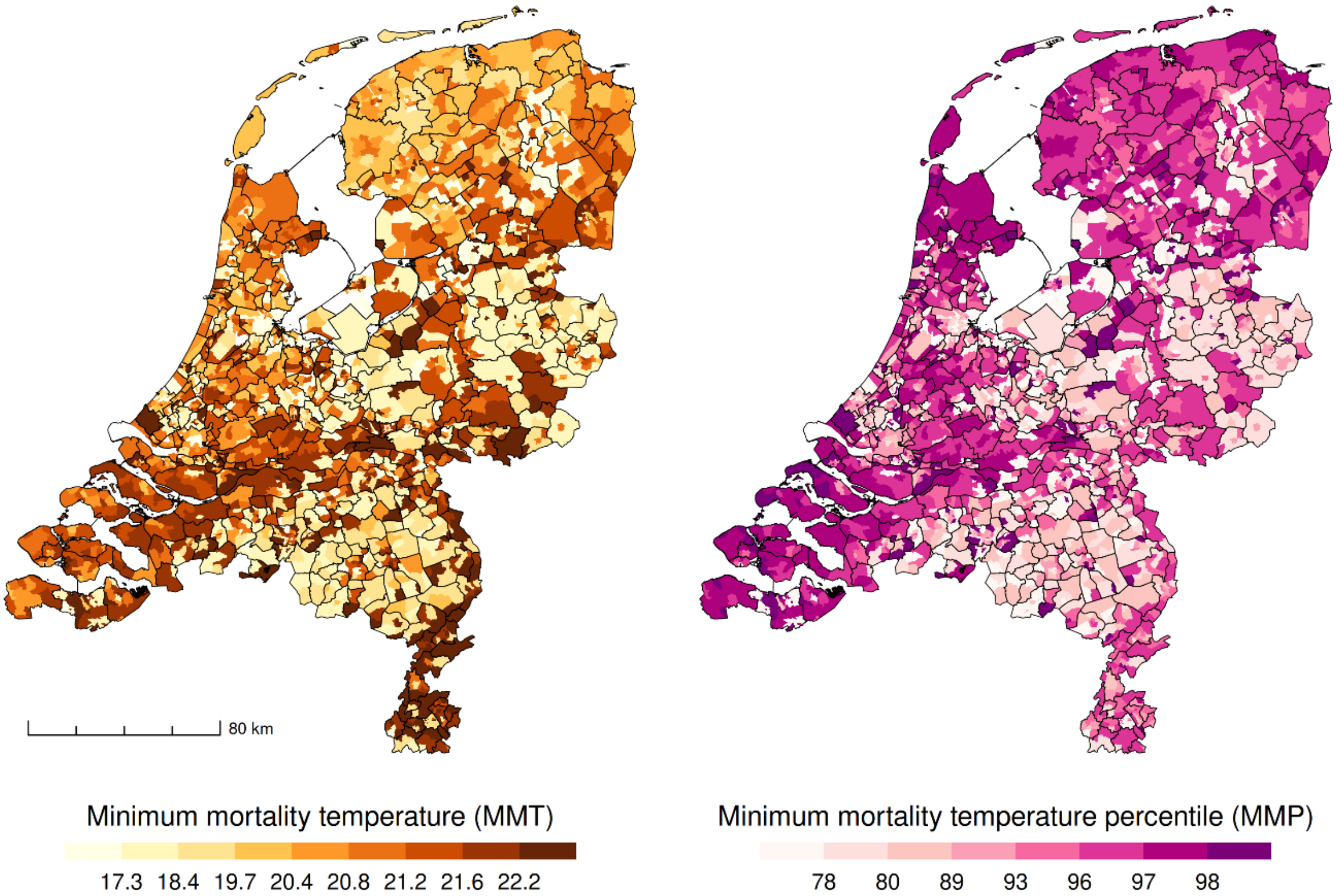

[3] The legend intervals are not equally spaced, but correspond to the 10th percentiles of the distribution of the MMT and MMP.

Figure S9. Geographical distribution of the relative risk (RR) associated with heat (99th temperature percentile vs MMT - left) and cold (1st vs MMT - right) by age group (younger in top, older in bottom figure) across districts in the Netherlands.[4]

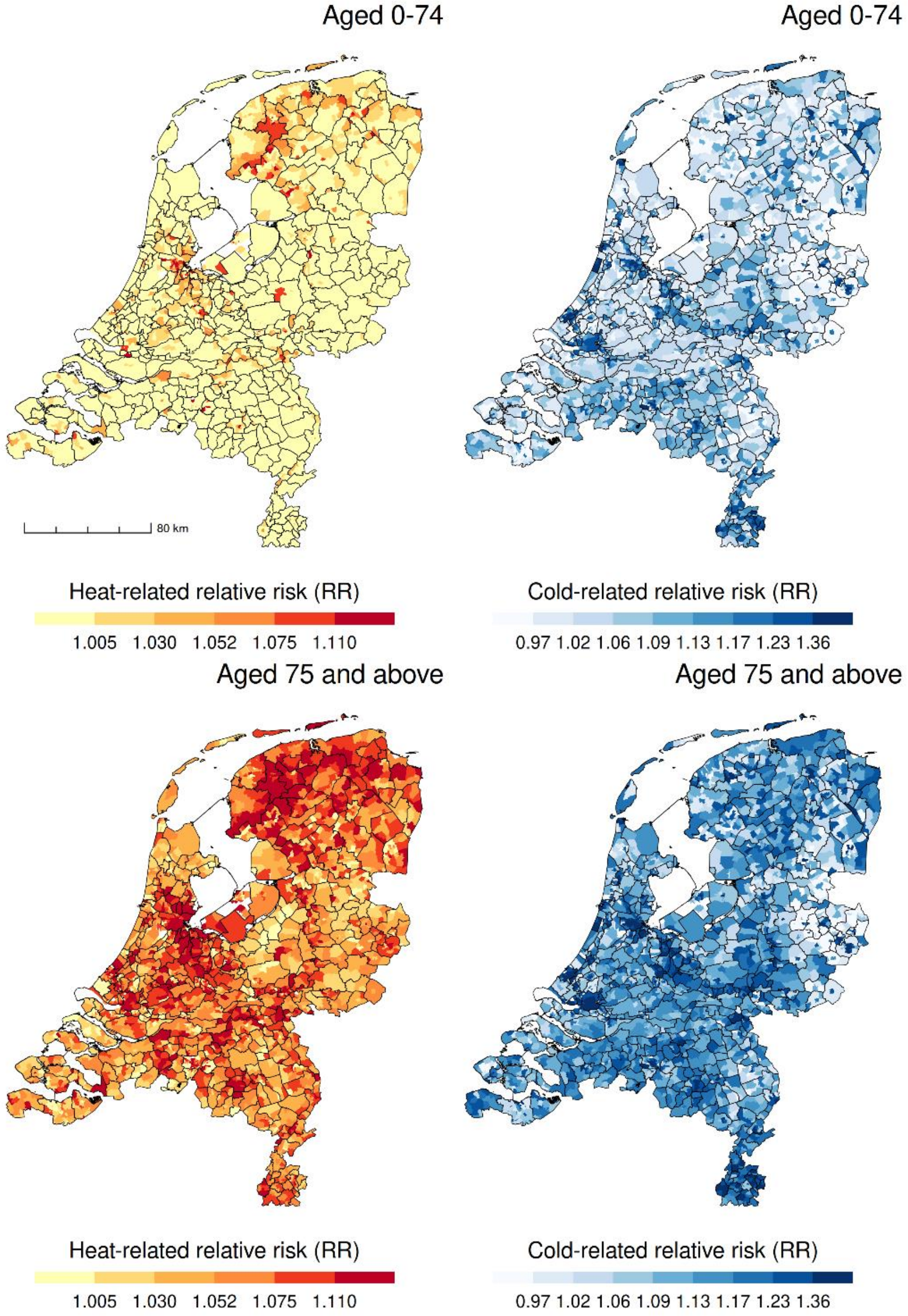

[4] The legend intervals are not equally spaced, but correspond to the 10th percentiles of the distribution of the RRs.

Table S5. Annual excess deaths and standardized excess mortality rates attributable to cold and heat in the Netherlands for the years 2010-2019.

**Annual excess deaths [CI]**		**Standardized excess mortality rate per 100,000 person-years [CI]**	
**Cold**	**Heat**	**Cold**	**Heat**
11,988 [7,554 – 15,609]	346[44 – 558]	73.33 [46.83 – 94.74]	2.19 [0.29 – 3.51]

Figure S10. Pooled estimates of the overall cumulative exposure-response relationships between temperature percentile and all-cause mortality by age group in the main model and four sensitivity analyses.

Main model = uses three knots (10th, 75th, 90th percentile) and a cubic B-spline to parametrize the exposure response function, and 21 days for the lag-response function.

Different knots = uses three knots (20th,70th,85th percentile) and a cubic B-spline to parametrize the exposure response function, and 21 days for the lag-response function.

Different spline function = uses three knots (10th, 75th, 90th percentile) and a natural spline to parametrize the exposure response function, and 21 days for the lag-response function.

Lag 0-2 days = uses three knots (10th, 75th, 90th percentile) and a cubic B-spline to parametrize the exposure response function, and 2 days for the lag-response function.

ERA5 temperature = uses ERA5 temperature as the exposure.

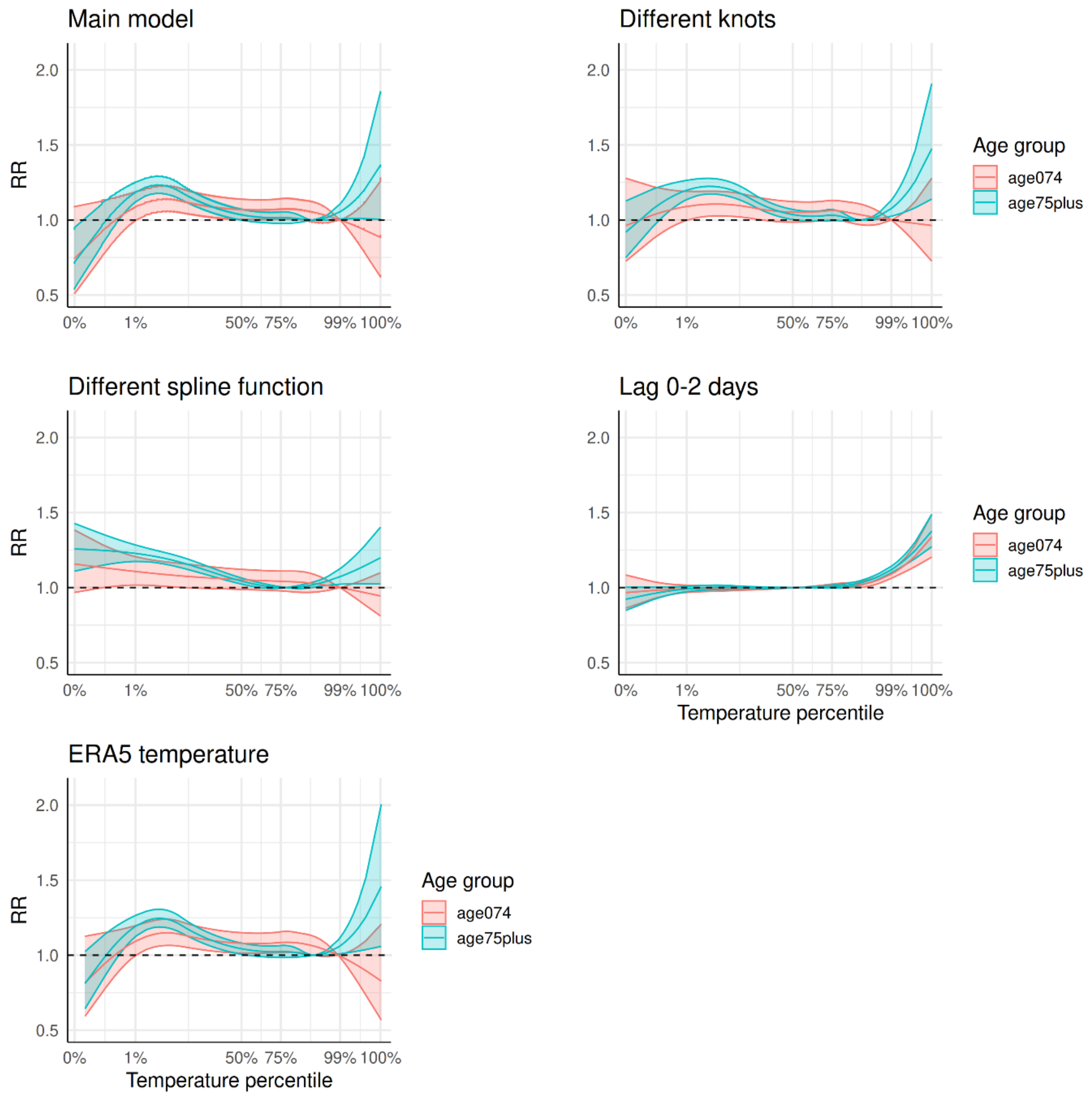

References:

1. CBS. Kerncijfers wijken en buurten 2019. In: Statistiek CBvd, editor. 2019.
2. Bussalleu A, Hoek G, Kloog I, Probst-Hensch N, Röösli M, de Hoogh K. Modelling Europe-wide fine resolution daily ambient temperature for 2003–2020 using machine learning. Science of the Total Environment. 2024;928:172454.
